# The Effect of X-Ray and Heavy Ions Radiations on Chemotherapy Refractory Tumor Cells

**DOI:** 10.3389/fonc.2016.00064

**Published:** 2016-03-29

**Authors:** Zhan Yu, Carola Hartel, Diana Pignalosa, Wilma Kraft-Weyrather, Guo-Liang Jiang, David Diaz-Carballo, Marco Durante

**Affiliations:** ^1^Department of Biophysics, GSI Helmholtzzentrum für Schwerionenforschung, Darmstadt, Germany; ^2^Department of Radiation Oncology, Shanghai Proton and Heavy Ion Center, Shanghai, China; ^3^Department of Oncology, Shanghai Medical College, Fudan University, Shanghai, China; ^4^Institute of Molecular Oncology and Experimental Therapeutics, Marienhospital Herne, Ruhr University of Bochum Medical School, Herne, Germany; ^5^Institute of Condense Matter Physics, Darmstadt University of Technology, Darmstadt, Germany

**Keywords:** chemoresistance, X-ray and heavy ion irradiation, relative biological effectiveness, neuroblastoma, glioblastoma

## Abstract

**Purpose:**

The purpose of this study is to link both numeric and structural chromosomal aberrations to the effectiveness of radiotherapy in chemotherapy refractory tumor cells.

**Materials and methods:**

Neuroblastoma (LAN-1) and 79HF6 glioblastoma cells derived from patients and their chemoresistant sublines were artificially cultured as neurospheres and irradiated by X-rays and heavy ions sources. All the cell lines were irradiated by Carbon-SIS with LET of 100 keV/μm. However, 79HF6 cells and LAN-1 cells were also irradiated by Carbon-UNILAC with LET of 168 keV/μm and Nickel ions with LET of 174 keV/μm, respectively. The effect of radiation on the survival and proliferation of cells was addressed by standard clonogenic assays. In order to analyze cell karyotype standard Giemsa staining, multicolor fluorescence *in situ* hybridization (mFISH) and multicolor banding (mBAND) techniques were applied.

**Results:**

Relative biological effectiveness values of heavy ion beams relative to X-rays at the D_10_ values were found between 2.3 and 2.6 with Carbon-SIS and Nickel for LAN-1 and between 2.5 and 3.4 with Carbon-SIS and Carbon-UNILAC for 79HF6 cells. Chemorefractory LAN-1^RETO^ cells were found more radioresistant than untreated LAN-1^WT^ cells. 79HF6^RETO^ glioblastoma cells were found more radiosensitive than cytostatic sensitive cells 79HF6^WT^. Sphere formation assay showed that LAN-1^RETO^ cells were able to form spheres in serum-free culture, whereas 79HF6 cells could not. Most of 79HF6^WT^ cells revealed a number of 71–90 chromosomes, whereas 79HF6^RETO^ revealed a number of 52–83 chromosomes. The majority of LAN-1^WT^ cells revealed a number of 40–44 chromosomes. mFISH analysis showed some stable aberrations, especially on chromosome 10 as judged by the impossibility to label this region with specific probes. This was corroborated using mBAND analysis.

**Conclusion:**

Heavy ion irradiation was more effective than X-ray in both cytostatic naive cancer and chemoresistant cell lines. LAN-1^RETO^ chemoresistant neuroblastoma cells were found to be more radioresistant than the cytostatic naive cells (LAN-1^WT^), whereas this effect was not found in 79HF6 cells.

## Introduction

There is convincing evidence that many solid and hematological malignancies are organized hierarchically and contain a small population of cancer stem cells (CSCs) that possess the capacity to self-renew and to cause the heterogeneous lineages of cells that form the tumor (1). Consequently, cell heterogeneity of tumors may play an important role in tumor persistence and metastasis formation. Additionally, there is growing evidence that CSCs are inherently resistant to radiation and perhaps other conventional anticancer treatments, i.e., chemotherapy (2–4). These intrinsic mechanisms of resistance are responsible for a significant number of tumor recurrences (2, 3). Consequently, an effective anticancer treatment can only be achieved if this population is eliminated.

Chemotherapy has the advantage over radiotherapy in fighting the disseminated metastatic situation but at higher costs for the organism as a whole. Contrary to that, radiotherapy is a more localized treatment, but it is less applicable once the cancer has spread to several regions. Contemporaneous studies have consistently shown that CSC phenotypes are triggered after chemotherapy courses with an accompanied radioresistance of cancer cells both *in vitro* and *in vivo* probably by preferential activation of the DNA damage response (5). This indicates the urgent necessity for reevaluation of conventional therapies and searching for new ones that focus on CSCs to enhance the efficacy of cancer treatments.

Neuroblastoma is one of the most common extracranial pediatric tumors with a wide spectrum of clinical forms. The long-term survival of children with a high-risk clinical phenotype is <40% (especially those with MYCN amplification) (6). Glioblastoma is the most aggressive brain tumor in adults. In spite of multimodal therapy, the median survival is only around 14 months with early recurrences (and infiltrative events) in the brain (7). The existence (and local spread) of CSCs may be an important reason for the treatment failure due to its resistance to conventional therapy, which leads to a poor prognosis.

Culturing cancer cells in the presence of a low dose of chemotherapeutic agents is one of the approaches to enrich subpopulations with CSC-like phenotypes and related physiology. Etoposide is a topoisomerase inhibitor and causes DNA breaks enforcing apoptosis in dividing cancer cells. It is used as a standard chemotherapy in many tumors, such as neuroblastoma. However, etoposide is also known as an inducing agent of multidrug-resistant cancer phenotypes. In this study, low dose of etoposide was used to enrich CSCs fraction in glioblastoma and neuroblastoma cell lines.

Particle radiotherapy is becoming more widely used because proton and heavy ions have a favorable depth–dose distribution and a higher relative biological effectiveness (RBE) compared with photon. Once cancer cells are exposed to this therapy, they suffer a complex and clustered DNA damage, which is unable to be repaired by cellular mechanisms independent of the reactive oxygen species formed after exposing cells to charged particles. Consequently, malignant cells are less radioresistance because the mechanisms responsible for DNA reparation work less effective (8).

Our works aimed at studying the survival of chemoresistant cells compared with their wild-type parentals after being exposed to X-rays and heavy ions. We also addressed the question if the karyotype and chromosomal number deviations are related to the survival.

## Materials and Methods

### Cell Lines and Culture Conditions

Two parental and their subtypes highly chemotherapy refractory cell lines LAN-1^WT^, LAN-1^RETO^ neuroblastoma and 79HF6^WT^, 79HF6^RETO^ glioblastoma multiforme derived from human tumors were used in this investigation. The LAN-1 cells were isolated from a bone marrow metastasis of a 2-year-old boy with neuroblastoma (clinical Stage IV), and the 79HF6 cells were isolated from a female adult patient. The etoposide-resistant sublines usyed in this work exhibit CSC features among a set of CSC markers, broad spectrum of cross-resistance to several cytostatics, and radioresistance. The phenotype characteristics and the CSC features were published previously (5). Cells were cultured in Dulbecco’s modified Eagle medium (DMEM), supplemented with 10% fetal calf serum (FCS) and 1% penicillin/streptomycin (all purchased from Biochrom AG, Berlin, Germany), and kept in a humidified atmosphere of 5% CO_2_ at 37°C. Resistant to ETOposide (RETO) cells were constantly cultured in the medium containing 4 μg/ml etoposide (Teva, Germany). The cell doubling time (*t*_D_) was determined in the exponential phase of the growth with the GSI in house program gd (©M. Krämer, 2003).

### Clonogenic Assay

Clonogenic assay was performed to determine both clonogenic behavior and cell survival rates after irradiation. Cells were seeded in T25 flask containing around 100 viable colonies after irradiation. LAN-1^WT^ and LAN-1^RETO^ cells were incubated for 9 days. 79HF6^WT^ cells were incubated for 11 days, whereas 79HF6^RETO^ cells were incubated for 25 days. Colonies were fixed and stained with methylene blue. Colonies containing more than 50 cells were defined as survivors.

### Sphere Formation Assay

Cells were cultured in serum-free neurobasal A medium (Gibco, Life Technologies, Germany) supplemented with B27 (Gibco, Life Technologies, Germany), 10 ng/ml human fibroblast growth factor-basic (Biochrom, Germany), 20 ng/ml human epidermal growth factor (Biochrom, Germany), and 0.1% bovine serum albumin fraction V (Roche Diagnostics, Germany) to observe the formation of neurospheres (9).

### Karyotyping

For chromosome preparations, cells were seeded 48 h in T75 culture flasks with 10 ml medium before the experiment in order to allow stabile attachment. One hundred microliters of colcemid (Roche Deutschland Holding GmbH, Germany) with the concentration of 10 μg/ml were added to the cultures. After 3.5 h of incubation for LAN-1 and 79HF6^WT^ and 4 h for 79HF6^RETO^, cells were trypsinized and harvested. Cell suspension was pelleted and carefully treated with prewarmed (37°C) 0.075M potassium chloride solution for 8 min and then fixed with 3:1 ratio of MeOH:glacial acetic acid for 30 min at room temperature. After washing, cells were resuspended in proper volume of the mentioned fixative and dropped on wet slides. The slides were then air-dried for 24 h. The slides were stained with 5% Giemsa (Merck, Germany) solution for 10 min, washed with distilled water, and dried overnight.

### Multicolor Fluorescence *In Situ* Hybridization Technique and Multicolor Banding Technique

For multicolor fluorescence *in situ* hybridization (mFISH) analysis, the slides were hybridized using the 24XCyte mFISH kit (Metasystems, Altlussheim, Germany) according to the protocol recommended by the manufacturer. In brief, the slides were first subjected to a denaturation followed by dehydration. An appropriate volume of DNA denatured probe was incubated in a humidified chamber at 37°C in the dark for 2 days. Afterward, the remaining hybridization probe was washed off. Finally, all DNA material was counterstained using DAPI/antifade (250 ng/ml), and the slide was covered. The chromosomal dispersal was analyzed using fluorescence microscopy Imager Z1 (Zeiss, Germany). Probes labeled with FITC, Orange, Texas Red^®^, Aqua, Cy^TM^5 (Cy5), and 4′,6-diamidino-2-phenylindol (DAPI) fluorochromes were used to visualize chromosomal segments. Karyotypes were (re)constructed using the Isis/mFISH software (Metasystems). The procedure of multicolor banding (mBAND) is similar to that of mFISH, performed with the mBAND kit (Metasystems, Altlussheim, Germany), as previously described.

### Ionizing Irradiation

All the irradiations were performed in GSI. The X-ray irradiation was carried out using an Isovolt DS1 X-ray machine (Seifert, Ahrensberg, Germany), exposing cells to 250 kVp and 16 mA.

Ion irradiation was performed in a synchrotron machine of the GSI. For irradiation at Carbon-SIS facility, cells were cultured in T12.5 culture flasks and were completely filled with culture medium before irradiation. The cells were irradiated with 10 mm spread out Bragg peak (SOBP) with LET of 100 keV/μm. All the cell lines were irradiated by Carbon-SIS. For experiments using a Carbon-UNILAC, 79HF6 cells were cultured in 3 cm Petri dishes and placed into compatible Petri dish magazines for irradiation. The carbon ions had a primary energy of 11.4 MeV/u and the energy decreased to 9.9 MeV/u when stopping on target with the corresponding LET of 168 keV/μm (10). After irradiation, the inner border of Petri dish was cleaned using sterile cotton to remove unirradiated medium accumulated at the bottom of the inner border, because dishes were irradiated in a vertical position. As survival of cells is related to the LET of the beam, we use those two carbon beams with different LETs. LAN-1 cells were also irradiated by Nickel ions with energy of 1 GeV/u and LET of 174 keV/μm, because the beam time is limited in GSI and we got the chance of irradiated by Nickel ions with similar LET to Carbon-UNILAC, which is a higher LET beam compared with Carbon-SIS.

Samples in triplicate were subjected to irradiation sections for each dose with X-ray, heavy ions, and repeated at least three times. The irradiation doses for LAN-1^WT^ were fixed from 0 to 7 Gy for X-ray, from 0 to 2 Gy for Carbon, and from 0 to 2 Gy for Nickel. However, the doses for LAN-1^RETO^ were from 0 to 9 Gy for X-ray, 0 to 3.2 Gy for Carbon, and 0 to 2 Gy for Nickel. The doses for 79HF6^WT^ and 79HF6^RETO^ were applied from 0 to 10 Gy for X-ray, 0 to 5 Gy for Carbon-SIS, and 0 to 2.72 Gy for Carbon-Unilac.

Cell survival curves of X-ray were fitted with the linear-quadratic model (Eq. 1):

(1) $$S=e^{\left(-\alpha \text{D}-\beta {\text{D}}^{2}\right)}$$

Cell survival curves of heavy ions were fitted with a pure exponential equation (Eq. 2):

(2) $$S=e^{\left(-\alpha \text{D}\right)}$$

RBE_10_ values were calculated at 10% of survival level according to Eq. 3:

(3) $${\text{RBE}}_{\text{1}0}={\text{D}}_{\text{1}0}\text{ X}-\text{ray}/{\text{D}}_{\text{1}0}\text{ ions}$$

All of the fitting was performed with the GSI in house program gd (©M. Krämer, 2003).

### Statistical Analysis

Experiments were performed at least in triplicate, and the survival fraction of cells was given as mean ± SD. Karyotype and mBAND figures were descriptive and therefore not statistically analyzed.

## Results

### Differential Growth Patterns of LAN-1 Neuroblastoma and Glioblastoma 79HF6 Cell Lines

**Figure 1:**
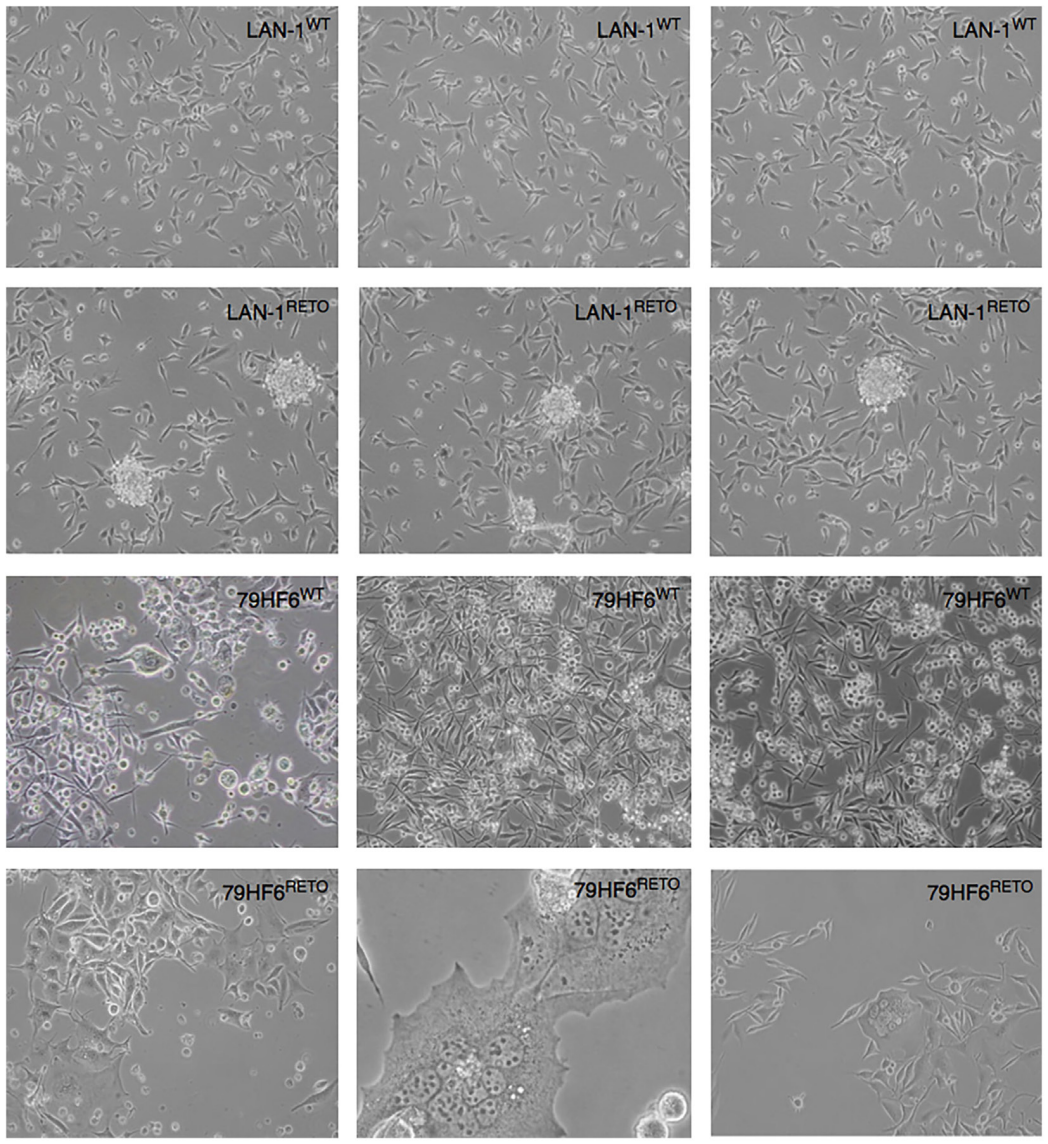
**Neurosphere-forming cells**. LAN-1^WT/RETO^ neuroblastoma cells and 79HF6^WT/RETO^ glioblastoma cells have shown different morphology in serum-free neurobasal A medium. Resistant LAN-1 subline showed the capacity to form neurospheres, whereas their wild-type parental cells are not able to form spheres. 79HF6^WT/RETO^ cells did not alter their growth pattern once incubated in this defined condition. Magnification: LAN-1, 10×; 79HF6^WT^, 10×; 79HF6^RETO^, 10×; central picture, 20×. Pictures are representative for different experiments.

All four cell lines, both wild type and resistant, grew adherently. The growth kinetic for all cells shows differential pattern as jugged by their doubling times. In this regard, the replication of LAN-1^WT^, LAN-1^RETO^, 79HF6^WT^, and 79HF6^RETO^ was observed at 21.7 ± 0.7, 16.9 ± 0.8, 21.6 ± 0.3, and 56.7 ± 5.2 h, respectively. LAN-1^WT^ could form spheres when cultured in serum-free neurobasal A medium, whereas the other tumor cells lines were not able to form neurospheres once cultured under this condition (Figure 1). LAN-1^RETO^ and 79HF6^RETO^ chemotherapy refractory cells are able to stably grow in the medium containing low concentrations of etoposide. The growth kinetic of LAN-1^RETO^ cells showed a faster cell replication than the wild-type parentals. Contrary to that, 79HF6^RETO^ cells grew slower than 79HF6^WT^. These dissimilar growth patterns are in part a consequence of the development of chemoresistance of both tumor entities, which are biochemically dissimilar.

### Effectiveness of Heavy Ion Irradiation in Comparison to X-Ray

As previously published by our group and others, resistance to etoposide induces radioresistance in both LAN-1 neuroblastoma and glioblastoma 79HF6 cell lines (5). To explore how heavy ion irradiation has advantages over the conventional X-ray exposures, we monitored the cell survival of these cells exposure to Carbon and Nickel ion irradiation.

**Figure 2:**
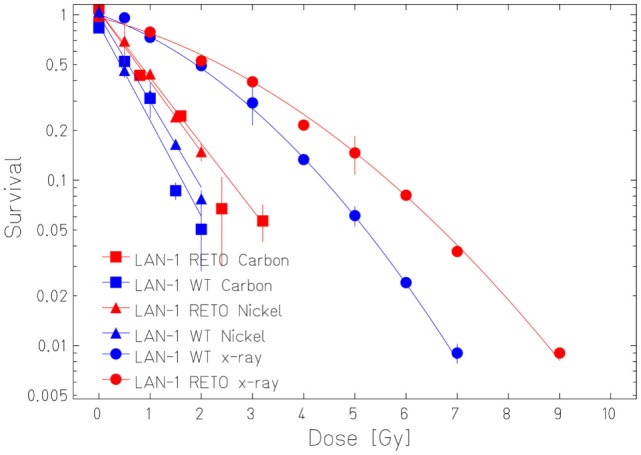
**Survival of LAN-1 neuroblastoma cells after ionizing irradiation**. LAN-1^WT/RETO^ cells were irradiated with X-ray, Nickel (174 keV/μm), and Carbon-SIS (100 keV/μm). Graphic depicts the results of three independent experiments. Points, the mean survival fractions; bars, SD.

**Figure 3:**
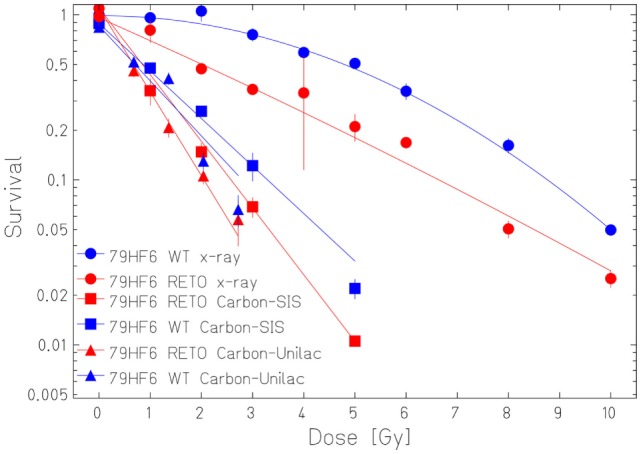
**Survival of 79HF6 glioblastoma cells after ionizing irradiation**. 79HF6^WT/RETO^ cells were irradiated with X-ray, Carbon-SIS (100 keV/μm), and Carbon-Unilac (168 keV/μm). Graphic depicts the results of three independent experiments. Points, the mean survival fractions; bars, SD.

The survival curves showed that heavy ion irradiation was more effective than X-ray in all four cell lines (Figures 2 and 3). The RBE values of heavy ions beam relative to X-rays at the D_10_ values were from 2.3 to 2.6 for LAN-1 cells and 2.5 to 3.4 for 79HF6 cells (Table 1). For LAN-1 cells, the etoposide-resistant subtypes (cultured in the presence of etoposide) were found to be more radioresistant than WT cells (cultured without etoposide) after X-ray and heavy ion irradiation (Figure 2), but for 79HF6 cells, RETO cells are more sensitive than WT cells after X-ray and heavy ion irradiation (Figure 3).

**Table 1:** **Relative biological effectiveness values of heavy ions in LAN-1 neuroblastoma and 79HF6 glioblastoma cells**.

Cell line	Carbon	Carbon-SIS	Carbon-Unilac	Nickel
LAN-1^WT^	2.60 ± 0.20	–	–	2.30 ± 0.20
LAN-1^RETO^	2.28 ± 0.09	–	–	2.42 ± 0.04
79HF6^WT^	–	2.50 ± 0.10	2.90 ± 0.20	–
79HF6^RETO^	–	2.70 ± 0.50	3.40 ± 0.20	–

*The relative biological effectiveness (RBE) values of heavy ions beam relative to X-rays at the D_10_ values were found between 2.3 and 2.6 for LAN-1 and 2.5 and 3.4 for 79HF6 cells, respectively*.

### Chromosomal Aberrations Found in LAN-1 Neuroblastoma and Glioblastoma 79HF6 Cell Lines

**Figure 4:**
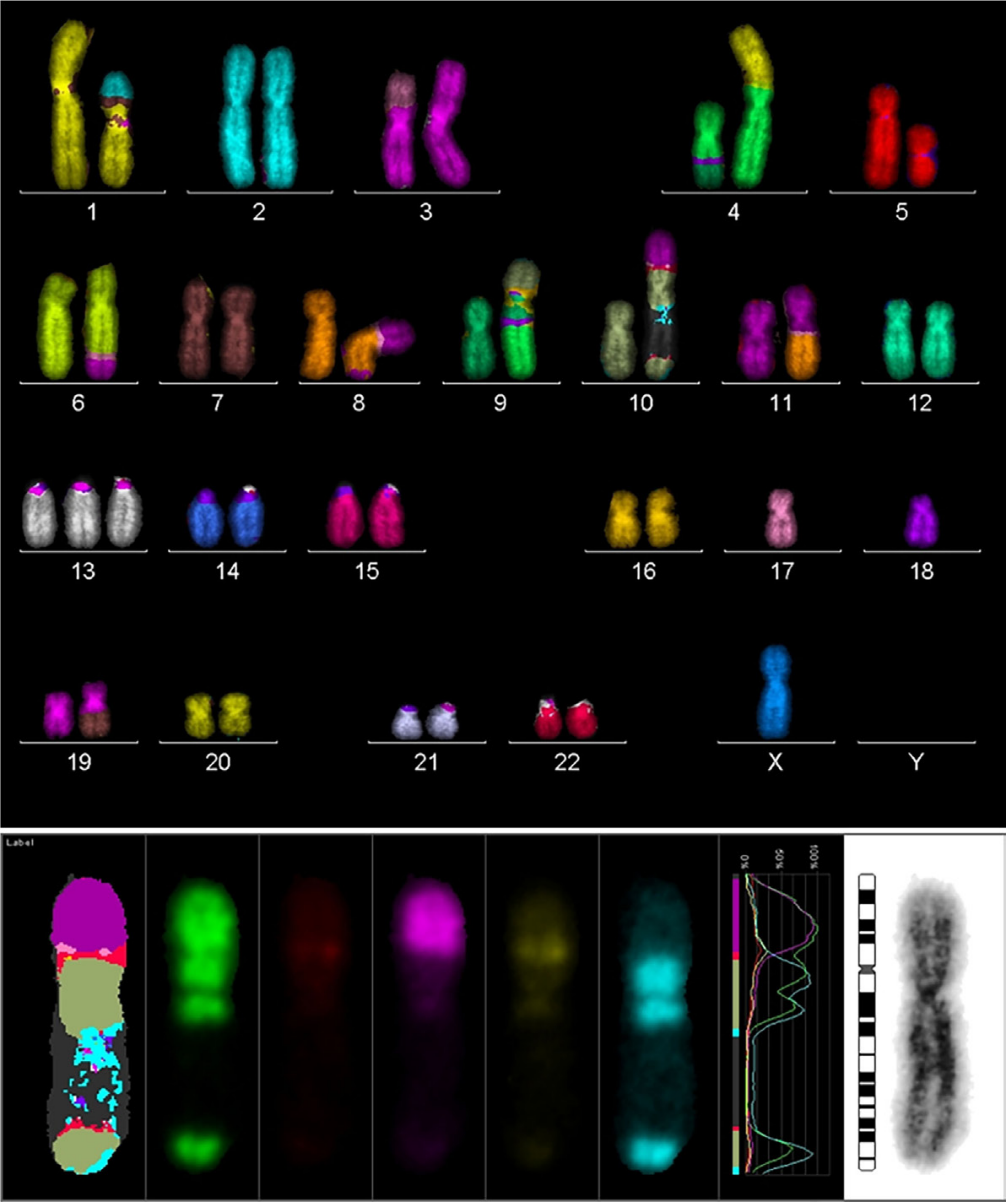
**Karyotype of LAN-1 neuroblastoma cells analyzed by mFISH**. LAN-1 cells revealed a range of 40–44 chromosomes per cell. Besides the segmental exchange between chromosomes, the most prominent observation in this cell line was an unstained region in chromosome 10. Structurally, chromosome 10 appeared to be intact (lower picture) once stained with Giemsa, but lower arms were not able to hybridize with the corresponding probes. Picture is representative for several cells analyzed under the same conditions. Magnification 100×.

**Figure 5:**
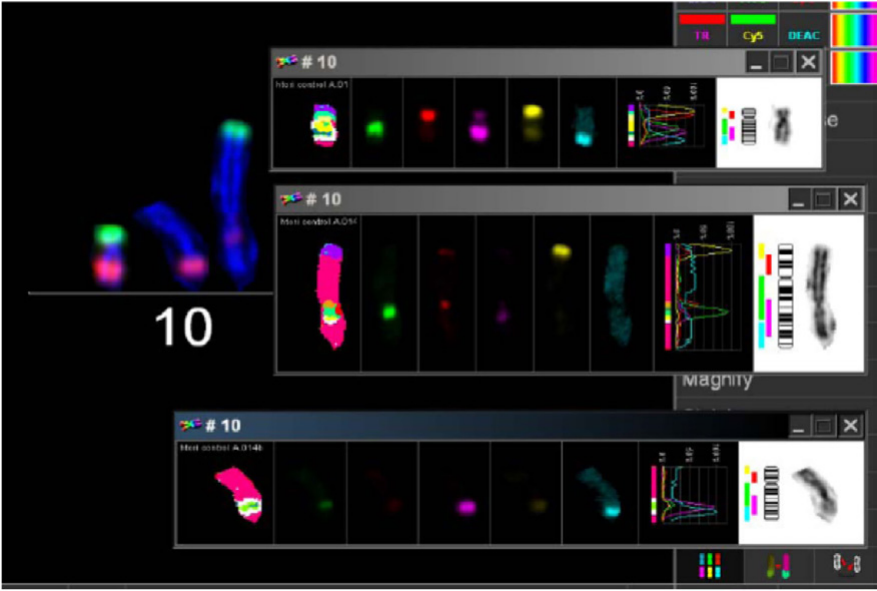
**mBAND analysis of chromosome 10 of LAN-1 neuroblastoma cells**. As detected in the karyotype, LAN-1 cells showed an unstained region that was localized on 10p. This arm was not able to hybridize with the corresponding probes directed toward this region. Picture is representative for several cells analyzed under the same conditions. Magnification 100×.

In order to search for the cause of radioresistance, we analyzed the karyotype of all cells used in our study. Most of LAN^−^ cells had 40–44 chromosomes, and mFISH showed some stable aberrations, especially on chromosome 10 with an unstained region (Figure 4), and mBAND showed the unstained region located on 10p (Figure 5).

**Figure 6:**
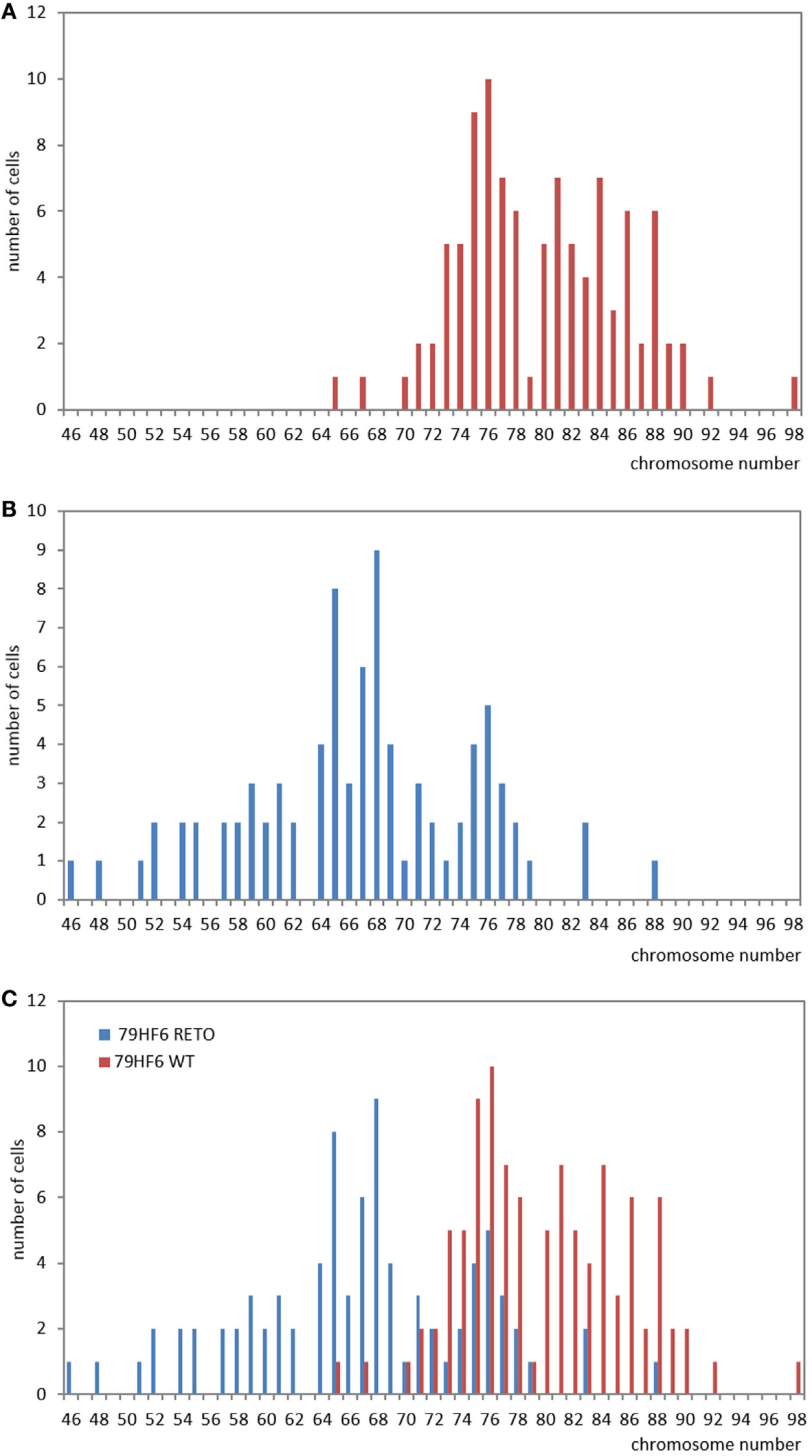
**Studies on chromosome number in 79HF6 glioblastoma cells**. 79HF6^WT/RETO^ glioblastoma cells revealed strong differences in their chromosome number. Wild-type cells showed polyploidy **(A)**. On the contrary, the resistant subline 79HF6^RETO^ showed less numerical aberrations **(B)**. Both diagrams were merged to provide an overall look of the chromosomal dispersion in both cells types **(C)**. These differences were found to be highly significant once applied a descriptive statistic. Graphics are representative for several experiments with the same experimental conditions.

The chromosomal number in 79HF6 glioblastoma cells enormously differed in the resistant subline. Most of the 79HF6^WT^ cells revealed a number of 71–90 chromosomes, whereas 79HF6^WT^ cells reflected 52–83 chromosomes. The chromosomal number of 79HF6^RETO^ revealed two peaks (Figure 6). It indicated that when cells were exposed to etoposide, the chromosome had the tendency to decrease to its relative normal ploidy. This phenomenon could be explained as the effort of tumor cells on maintaining gene stability. Diploid chromosomal distribution is more stable compared with polyploidy numbers, especially when tumor cells suffer the injury of chemical agents or other stressors. Tumors cells carrying a polyploidy derived a subgroup with a more stable karyotype in order to maintain the gene stability.

## Discussion

Cancer stem cells show continuous self-renewal, extensive parenchymal migration/infiltration, and potential for full or partial differentiation in all cell types, which constitute a tumor. To explore how LAN-1 neuroblastoma and glioblastoma 79HF6 cell lines growth, we cultured these cells under optimal conditions for propagation in serum-free neurobasal A medium. It is known that under these conditions, cells displayed profound biological differences in growth patterns and were enforced to grow as non-adherent, multicellular spheres, inducing CSC-like populations (9).

Every cell type obviously showed different morphologies, as they were cultured in serum-free medium or serum-contained medium. Sphere formation assay (11, 12) performed to select CSC phenotypes from both cell types revealed that LAN-1^RETO^ cells were able to form neurospheres after culturing them in serum-free medium, instead LAN-1^WT^, 79HF6^WT^, and 79HF6^RETO^ cells were not capable to form neurospheres under the same conditions. CSCs may have the competence of durable self-renew, the capacity to develop and maintain tumor-related cell heterogeneity, differentiation, as well as the ability of both radioresistance and chemoresistance. To confirm the existence of CSC features, cells were transplanted to hosts and expected to induce tumor and maintain the features of parental tumor cells (13, 14). Studies in the past (5) have shown that both cell lines have CSC-like features, including chemoresistance and radioresistance to X-ray.

Our studies revealed that heavy ions had higher cell killing efficiency in both neuroblastoma and glioblastoma cell lines, despite its chemoresistance and chromosomal normality status. 79HF6^WT^ cells were very resistant to X-ray. The survival rates of these cells were nearly not affected with 1 or 2 Gy of exposure to X-ray and were still around 5% with 10 Gy. This could explain why glioblastoma is so hard to be treated in clinic with conventional X-ray. However, RBE_10_ was 2.5–2.9, which indicates that heavy ions had notable advantages in killing radioresistant tumors as glioblastoma.

Our studies also revealed that LAN-1^RETO^ cells were found more radioresistant to X-ray than LAN-1^WT^. Contrary to that, 79HF6^RETO^ glioblastoma cells were less radioresistant than its wild-type parentals. The probable reason for this difference could be the different growth rates or the chromosomal number (15, 16). 79HF6^RETO^ revealed a high variation in number of chromosomes in comparison to the wild-type cells that are more homogeneous. Thus, cells with a chromosomal abnormality in number are more sensible to ion irradiation. Although LAN-1^RETO^ cells were made more resistant to radiation and were able to form neurospheres after exposure to etoposide, the opposite was true for 79HF6^RETO^. This may imply the higher level point that general CSC features are enhanced by etoposide in LAN-1 and CSC features may be reduced by etoposide in 79HF6. This difference could be caused by the inherent biological difference of neuroblastoma and glioblastoma. It may also be related to the concentration of etoposide. When the concentration is different, the results could change. Clearly, more work is needed to find the reason.

The biological hallmark of neuroblastomas is the complexity of the genetic abnormalities developed by the tumor cells, which are powerful prognostic markers. The most consistent abnormalities found in this tumor entity include ploidy changes, deletions of chromosome arms, amplification of the MYCN oncogene, and most frequently gains of chromosome arm 17q (6). There was a region in LAN-1 cells, which could not be stained by any fluorescence after mFISH procedure on chromosome no. 10. The analysis of mBAND corroborated that the unstained region was located on chromosome arm 10p. Homogeneously staining regions (HSRs) were localized on chromosome arm 10p and 10q of neuroblastoma cells with G-banding technique (17). HSR was cytogenetic evidence of a probable gene amplification. The unstained region of mFISH and mBAND of LAN-1^WT^ cells was probably caused by repeated gene amplification or very short gene sequences. When gene segments are shorter than the probes of mFISH and mBAND, the fluorescently labeled probes could not properly anneal with the complementary sequences. Because of this, chromosome arm 10p showed an unlabeled region.

Recurrent loss of genetic material is normally found on chromosome arms 3p, 10p, 10q, 16q, and 20q in the hereditary neuroblastomas, in addition to regions usually deleted in sporadic neuroblastomas (1p36 and 11q) (18). These chromosomal sites may harbor tumor suppressor genes. Furthermore, loss of heterozygosis (LOH) at chromosome 10 is found exclusively at 10p11.23–p15.1 and consequently associated with MYCN-amplified Stage IV tumors in neuroblastoma tumors (19). LOH could induce different changes in one pair of alleles on certain gene and loss of part or even whole gene sequence of the allele. Usually, LOH is commonly related to the deficiency of tumor suppressor genes, i.e., p53. In the case where two alleles exist, the tumor suppressor gene will suppress the generation of tumors. In normal cells, when one allele is abnormal or lost, this defective suppression drive cells to immortalization. Thus, chromosome 10p probably encloses certain tumor suppressor genes. In our study, chromosome 17 reflects a deletion. Considering that p53 gene is located on this chromosome, the lack of p53 gene due to LOH induces a defeat of tumor suppressor functions in these cells. Previous studies showed that tumor suppressor genes, i.e., p53 and so on, could regulate cell survival and death (20, 21). Chromosomal aberrations in these cells subsequently affecting tumor suppressor gene expressing could influence the survival of cells.

In summary, heavy ion irradiation is more effective than X-ray for both untreated and chemoresistant tumor cell lines. For LAN-1 cells, the chemoresistant subpopulation LAN-1^RETO^ is definitely more radioresistant than untreated cells (WT), while this effect was not found in 79HF6 cells.

## Author Contributions

ZY: substantial contributions to the conception of the work, performing the experiments, analysis, and interpretation of data for the work and drafting the work. CH: substantial contributions to the conception of the work, performing the experiments, analysis. DP: substantial contributions to performing the experiments, analysis. WK-W: substantial contributions to the conception of the work, revising it critically for important intellectual content. G-LJ: substantial contributions to the conception of the work, revising it critically for important intellectual content. DD-C: substantial contributions to the conception of the work, revising it critically for important intellectual content. MD: substantial contributions to the conception of the work, revising it critically for important intellectual content.

## Conflict of Interest Statement

The authors declare that the research was conducted in the absence of any commercial or financial relationships that could be construed as a potential conflict of interest.
